# Diversity of bacterial community in Jerusalem artichoke (*Helianthus tuberosus* L.) during storage is associated with the genotype and carbohydrates

**DOI:** 10.3389/fmicb.2022.986659

**Published:** 2022-09-15

**Authors:** Guolian Du, Zhu Sun, Shanhua Bao, Qiwen Zhong, Shipeng Yang

**Affiliations:** ^1^Qinghai Key Laboratory of Vegetable Genetics and Physiology, Agriculture and Forestry Sciences Institute of Qinghai University, Qinghai University, Xining, China; ^2^Laboratory for Research and Utilization of Germplasm Resources in Qinghai Tibet Plateau, Qinghai University, Xining, China

**Keywords:** Jerusalem artichoke, storage, microbiome, inulin, high-performance liquid chromatography, high-performance ion-exchange chromatography

## Abstract

Jerusalem artichoke (JA) is a fructan-accumulating crop that has gained popularity in recent years. The objective of the present study was to determine the dynamics of the JA-microbiome during storage. The microbial population on the surface of the JA tuber was determined by next-generation sequencing of 16S rRNA amplicons. Subsequently, the changes in carbohydrate and degree of polymerization of fructan in tubers during storage were measured. Among different genotypes of JA varieties, intergeneric differences were observed in the diversity and abundance of bacterial communities distributed on the surface of tubers. Additionally, bacterial diversity was significantly higher in storage-tolerant varieties relative to the storage-intolerant varieties. Redundancy analysis (RDA) and the correlation matrix indicated a relationship between changes in the carbohydrates and microbial community succession during tuber storage. The tuber decay rate correlated positively with the degree of polymerization of fructan. Moreover, *Dysgonomonas* and *Acinetobacter* in perishable varieties correlated significantly with the decay rate. Therefore, the bacteria associated with the decay rate may be involved in the degradation of the degree of polymerization of fructan. Furthermore, *Serratia* showed a significant positive correlation with inulin during storage but a negative correlation with the decay rate, suggesting its antagonistic role against pathogenic bacteria on the surface of JA tubers. However, the above correlation was not observed in the storage-tolerant varieties. Functional annotation analysis revealed that storage-tolerant JA varieties maintain tuber quality through enrichment of biocontrol bacteria, including *Flavobacterium*, *Sphingobacterium*, and *Staphylococcus* to resist pathogens. These results suggested that crop genotype and the structural composition of carbohydrates may result in differential selective enrichment effects of microbial communities on the surface of JA varieties. In this study, the relationship between microbial community succession and changes in tuber carbohydrates during JA storage was revealed for the first time through the combination of high-throughput sequencing, high-performance liquid chromatography (HPLC), and high-performance ion-exchange chromatography (HPIC). Overall, the findings of this study are expected to provide new insights into the dynamics of microbial-crop interactions during storage.

## Introduction

Complex carbohydrates, in the form of structural and storage polysaccharides, are products of primary metabolism. Biomass carbohydrates are a ubiquitous source of energy for microorganisms in different ecosystems (Gunina and Kuzyakov, 2015). Crop fruits and seeds are rich in carbohydrates, and their utilization by microorganisms is inextricably linked to different environmental factors and crop species. In the case of limited nutrient pools and changing temporal gradients, intense competitive relationships between microbial communities are found, which strongly influence the proliferation and survival of exogenous bacteria on agricultural products and changes with external conditions (Droby et al., 2016). Post-harvest fruit and vegetable loss accounts for nearly one-third of the total and majorly occurs during the processing and storage of agricultural products (Spang et al., 2019). Fruit decay crucially affects crop storage, processing, and merchantability (Wenneker and Thomma, 2020). Microbial communities on the surface of crop fruits are closely related to their decay. In addition, the equilibrium between microorganisms and their hosts is disrupted with increased storage time. The stability between the two is maintained through a complex molecular signaling system comprising microbial diversity and multiple factors. In addition, microbial diversity is associated with fruit quality (Zhang et al., 2021). Studies on bacterial diversity for varieties with different storage characteristics in the same environment can facilitate the understanding of fruit as an ecosystem, wherein bacterial communities play a crucial role in maintaining/regulating the health and physiology of fruits during the storage (Droby and Wisniewski, 2018).

Advances in next-generation sequencing (NGS) technologies have led to significant improvements in examining microbial communities. Several studies confirm that microorganisms identified by traditional isolated culture methods represent only 0.1–10% of the total environmental organisms (Amann et al., 1995; Tholozan et al., 1999). High-throughput sequencing allows for the extraction of DNA directly from different samples for the analyses of species composition and abundance without the need to isolate and culture them. Accumulating evidence from these techniques suggests that many microbial communities do not undergo spatial homogenization but display significant structures like many plant and animal communities. These findings are largely based on the analyses of bacteria (Morrison-Whittle and Goddard, 2015). Furthermore, these studies have focused on the following aspects: (1) specific microbial communities showing differential species composition and abundance among varieties of different crops (Zheng et al., 2015; Buchholz et al., 2021); (2) changes in microbial abundance over time during storage (Shen et al., 2018; Liu et al., 2020); (3) inhibitory effects of non-pathogenic microorganisms (Magerl et al., 2008), and (4) correlation with specific carbohydrates (Chatellard et al., 2016; Li et al., 2018). Although existing research extends to a wide range of crops, the diversity, function, and impact of specific communities of these communities on the overall community remain unknown. Consequently, strategies to prolong shelf life and avoid fruit diseases during storage should account for the indigenous microbiome and strategies should be implemented for a sustainable management (Kusstatscher et al., 2020).

Jerusalem artichoke (JA) is a perennial herb of the genus, *Helianthus* L., belonging to Asteraceae. Inulin, primarily stored in the tuber, accounts for 80% of the total dry weight of JA. Inulin-type fructans (ITFs), which are functional fructans and soluble dietary fibers, comprise a mixture of inulin, oligofructose, and fructooligosaccharide with β configuration. ITFs are mainly distributed in monocotyledonous and dicotyledonous plants, including JA, chicory, asparagus, wheat, garlic, banana, and onion (across Asteraceae, Gramineae, and Liliaceae). Inulin can regulate intestinal flora and is commonly used as a sugar substitute among patients with obesity, diabetes, and hyperlipidemia (Lovegrove et al., 2017; Man et al., 2020). ITF can be further classified into three subclasses of fructose units based on chain length or degree of polymerization (DP) as follows: small (3–5), medium (6–10), and long-chain (11–60). The length of DP is determined by genotype, environmental factors, harvest time, and storage processes. In general, DP determines the quality of the ITF and its commercial price. The type of carbohydrate determines to an extent the enrichment characteristics in microorganisms. Therefore, monitoring the development of bacterial communities during JA storage is crucial for understanding the primary pathogenic bacteria, improving the conservation of germplasm, and controlling fructan degradation.

In this study, we used high-throughput sequencing technology to analyze the bacterial community on the surface of tubers of jicama resources with different storage tolerance during storage. We focused on the following aspects: (1) decay-induced bacterial community changes between two different JA varieties with distinct storage-tolerant properties during storage to elucidate the correlation between microbial community structure and disease occurrence; (2) cluster heatmap and radar chart analyses of highly abundant bacterial communities. Clustering analysis was performed for bacterial communities to assess the microbial enrichment in JA during storage, while the radar chart revealed dynamic changes among the common bacterial communities; (3) RDA and correlation analysis of fructans and bacterial communities to screen key microbial factors showing high correlation with disease occurrence and to elucidate the variability in pathogenicity and disease suppression exhibited at different storage periods. Furthermore, it provides theoretical support for deciphering the microbial mechanisms involved in JA tuber storage rot, as well as for preventing and controlling JA tuber storage rot.

## Materials and methods

### Sample sources

#### Test samples and storage conditions

The JA resources, including the storage-tolerant variety ‘JA25’ and the storage-intolerant variety ‘JA187’, were provided by the Institute of Horticulture, Qinghai Provincial Academy of Agriculture and Forestry Sciences. Microbial samples on the surface of tubers were collected from the JA storage base (at a constant temperature of 2°C and relative humidity of 40–60%) of the Institute. According to previous statistics, the peak of cellar storage-induced decay rate was 68% in 2020.

#### Grading storage-induced tuber decay

The decay rate was defined as the proportion of tubers with visible signs of decay and color changes on the surface relative to the total number of tubers in each treatment group. The incidence and condition index were used as indicators to investigate the decay rate of stored JA tubers. In addition, the storage-induced tuber decay was classified into four classes according to the criteria listed in Table 1.

**Table 1:** Grading criteria for rotting disease in stored Jerusalem artichoke tubers.

Grading	Tuber presentation
0	No significant changes in tubers, with the absence of pathogens.
1	Slight damage with white mycelium appearing on the epidermis, accounting for less than 5% of the total surface area of the tuber.
2	Moderate damage with mycelium accounting for less than 6–25% of the total surface area and initial decay on of the tuber epidermis.
3	Severe damage with mycelium accounts for less than 26–50% of the total surface area and initial decay of the tuber flesh.
4	Severe tuber decay with the white mycelium turning dark gray, accounting for more than 75% of the total surface area of the tuber.

#### Microbial sample collection from the tuber surface

Based on the results of the decay estimation, the first microbial sample collection on the surface of tubers was performed on December 31, 2019, for Grade 1. The time interval between two sample collections was set at 30 days. Four critical sampling periods were determined based on the disease incidence. Microbial samples of tubers were collected from the storage-tolerant variety, ‘JA25’, and the storage-intolerant variety, ‘JA187’, during storage (six replicates per storage period and six randomly selected tubers per replicate). In addition, the microbial samples from the five tubers were pooled and packed in sterile valve bags after removing soil and other impurities and frozen in liquid nitrogen until subsequent use.

#### Preservation and processing of microbial samples

First, the mycelium on tuber surfaces was gently scraped with a sterile blade and placed into sterile containers. Subsequently, phosphate belanced solution (PBS) buffer was added to each of the 48 bacterial samples, mixed by shaking for 20 min, and centrifuged at 12,000 rpm for 10 min to obtain the precipitate. Next, the samples were loaded into centrifuge tubes and frozen in liquid nitrogen. Subsequently, the frozen tissues were stored at-80°C for DNA extraction and bacterial community analyses. Samples were labeled as ‘JA25’.1, ‘JA25’.2, ‘JA25’.3, ‘JA25’.4, ‘JA187’.1, ‘JA187’.2, ‘JA187’.3, and ‘JA187’.4 (corresponding to the four collection periods of the two sampled varieties) and sent to Beijing Novogene Biotech for 16 s rRNA sequencing. In addition, the remaining JA tubers were sliced, oven-dried, and packed in sterile valve bags for the determination of physicochemical properties, including the fructan content and DP.

### Determination of physicochemical properties of tuber carbohydrates

#### Fructan extraction and determination of its content

Distilled water (20 ml) was added to 0.5 g of dried JA powder and boiled in a water bath for 30 min, during which the samples were stirred several times using a glass rod. Next, the samples were allowed to cool at room temperature and poured into a centrifuge tube, and centrifuged for 10 min (12,000 r/min). Finally, the supernatant was poured into a 25 ml volumetric flask for determining the volume. Further,17.5 ml of distilled water and 1 ml of hydrochloric acid (3 mol L^−1^) were added to an equal weight of the dried JA powder and boiled for 1 h; the samples were stirred several times using a glass rod. Next, the samples were allowed to cool at room temperature. Subsequently, 1 ml of sodium hydroxide (3 mol L^−1^) and 0.6 ml of aluminum sulfate (3 mol L^−1^) were added to each sample, mixed well, poured into a centrifuge tube, and centrifuged for 10 min (12,000 r/min). Finally, the supernatant was poured into a 25 ml volumetric flask for determining the volume. Next, 1 ml of each solution from the reserve was added to 50 μl of sulfosalicylic acid (0.2 g L^−1^) and filtered through a filter membrane (0.22 μm). In addition, the samples were analyzed by HPLC with the following parameters: detector: differential refractive index detector; analytical column: SUGAR KS-802 (8.0 mmId × 300 mml) special column for sugar analysis; mobile phase: ultrapure water; flow rate: 1 ml/min; column temperature: 80°C, and injection volume: 5 μl.

Glucose, sucrose, and fructose standards were prepared (0.1, 0.25, 0.5, 0.75, and 1 mg/ml standard solutions). Subsequently, the samples were analyzed by HPLC (Shimadzu RID-10A, Kyoto, Japan), and the appearance periods were 7.12, 8.23, and 9.75 min for sucrose, glucose, and fructose, respectively. Finally, a linear graph was plotted based on the peak areas and the concentrations of the standards, the results of their linear regression equations are shown in Supplementary Table S1.

#### DP determination for fructan

The DP of inulin extracts was analyzed by high-efficiency ion chromatography coupled with pulsed amperometric detection (Au, Ag/AgCl reference electrodes). The inulin extracts were diluted to an appropriate concentration with deionized water (900 μl of ultrapure water was added to 100 μl of fructan extract), and filtered through a 0.22 μm filter membrane before injection. The sample was injected using an AS50 autosampler with the column temperature set at 30°C, the injection volume set at 5 μl and the flow rate set at 1 ml/min. A high-capacity anion-exchange column compatible with gradient elution was used for a set time of 60 min. The peak periods were 3.5, 4.6, 8.2, 13.6, 16.6, 19.1, 21.3, and 23.3 for 1-kestose, nystose, 1F-fructofuranosyl nystose, 1,1,1,1-kestohexose, fructoheptasaccharide, fructo-oligosaccharide DP8, fructo-oligosaccharide DP9, and fructo-oligosaccharide DP10, respectively. The relative percentage DP composition of inulin was calculated based on the peak area under the chromatogram, integrated with the Chromeleon^™^ software (version 6.2, Dionex).

### Analyses of the bacterial communities

#### Total DNA extraction

Genomic DNA of the samples was extracted using cetyltriethylammnonium bromide (CTAB) lysis buffer. Subsequently, the purity and concentration of DNA were measured by agarose gel electrophoresis (0.8%). An appropriate amount of sample DNA was taken in a centrifuge tube and diluted to a final concentration of 1 ng/μl in sterile water.

#### PCR amplification of the 16S rDNA-V4 region of the bacterial genome

Using the diluted genomic DNA as a template, the specific primers, 515F (5’-GTTTCGGTGCCAGCMGCCGCGGTAA-3′) and 806R (5’-CAGATCGGACTVGGGTWTCTAAT-3′), with barcodes for the 16S rDNA-V4 region were used according to the selected sequencing region. The 20 μl reaction system used to identify bacterial diversity was as follows: 5xFastPfu Buffer: 4 μl, 2.5 mm dNTPs: 2 μl, 515F (5 μm): 0.8 μl, 806R (5 μm): 0.8 μl, FastPfu Polymerase: 0.4 μl, BSA: 0.2 μl, template DNA: 10 ng, and ddH2O: 20 μl. In addition, the polymerase chain reaction (PCR) reaction parameters were as follows: pre-denaturation at 95°C for 3 min, denaturation at 95°C for 30 s, annealing at 55°C for 30 s, extension at 72°C for 45 s (27 cycles in total), and final extension at 72°C for 10 min. Finally, PCR amplification was performed using a high-efficiency and high-fidelity enzyme (Phusion^®^ High-Fidelity PCR Master Mix with GC Buffer, New England Biolabs) to ensure efficiency and accuracy of amplification.

#### Mixing and purification of PCR products

Mixing to equal concentration was performed according to the PCR product concentration, following which the PCR products were detected on 2% agarose gel at 120 V for approximately 30 min. Finally, the target bands were recovered using a gel recovery kit (Qiagen).

#### Library construction and sequencing

The purified PCR products were used to construct the library using the TruSeq^®^ DNA PCR-Free Sample Preparation Kit. The constructed libraries were quantified by Qubit and qPCR. Subsequently, the qualified libraries were sequenced on the NovaSeq 6,000 on the Illumina HiSeq 2,500 sequencing platform of Novogene.

#### Processing of sequencing data

In general, some noise is present in the raw data obtained after sequencing. Consequently, splicing and filtering were performed to obtain valid data for accurate and reliable analyses. First, data for each sample was split from the data to be sequenced according to barcode sequences and PCR amplification primer sequences. Next, the barcode and primer sequences were truncated. Subsequently, the reads in each sample were spliced using the FLASH software package (FLASH v1.2.7^1^; Magoč and Salzberg, 2011), and the resulting spliced sequences were the raw tags. Further, the raw reads were filtered using Qiime (V1.9.1; Caporaso et al., 2010) to obtain high-quality reads. Following the tag quality control process, the final valid data (effective Tags) were obtained after removing chimeric sequences (Rognes et al., 2016).

#### OTU clustering and species annotation

Operational Taxonomic Units (OTU) clustering was performed for the valid data at the 97% level using the Uparse software (Uparse v7.0.1001^2^; Haas et al., 2011). In addition, sequences with the highest frequency of occurrence of OTUs were screened as the representative sequences for those OTUs based on the ribosomal database project (RDP) classifier Bayesian algorithm using the Qiime (V1.9.1).^3^ Subsequently, species annotation analysis (with a threshold of 0.8 to 1) was performed based on the Mothur method using the small-subunit rRNA (SSUrRNA) database (Wang et al., 2007) from SILVA132 (Edgar, 2013). The structural composition of microbial communities was detected by statistical analysis of OTUs for abundance, α-diversity, β-diversity, and community outcomes for the species at each taxonomic level (DeSantis et al., 2006; Wang et al., 2007). In addition, the optimal comparisons were selected to determine the taxonomic information and species-based distribution of bacterial sequence abundance. Finally, the least amount of data among the samples were standardized for homogenization.

#### Sample complexity analysis

Sample complexity analysis was performed by assessing the alpha diversity (within-habitat diversity) and sample diversity indices were calculated using the Qiime software (Version 1.9.1). The ACE and Chao1 indices were used to evaluate community richness. Subsequently, community diversity was calculated using Shannon and Simpson indices. The sequencing depth index was assessed by Good’s coverage. Box plots between microbial community groups based on these diversity indices were drawn in R. Tukey’s HSD was used to examine the differences in microorganisms between different JA varieties during the storage period. Furthermore, effective tags were visualized using Origin 2019. Finally, the variance test was performed using Duncan’s new multiple range method using the SPSS software.

#### Multi-sample comparisons

Differences in bacterial species diversities between samples were compared using the ß-diversity index. Subsequently, Unifrac distances were calculated using the Qiime (Caporaso et al., 2010) software. Venn analysis was performed using the “VennDiagram” package in R software, which automatically generates highly-customizable, high-resolution Venn diagrams to assess common and unique microorganisms between JA varieties with differential storage characteristics across storage periods. In addition, ggplot2, reshape2, ggalluvial (Brunson, 2018), and vegan packages in R software were used to create collision diagrams of changes in microbial time series composition to visualize abundances of dominant bacterial and fungal taxa at phylum and genus levels. Subsequently, the top 35 bacterial genera in terms of abundance in each period were selected based on the abundance information for the bacterial communities, and clustering heatmaps were generated. Next, the common OTUs between the two varieties were selected to draw the radar chart. Finally, Linear discriminant analysis Effect Size (LEfSe) analysis was performed to identify changes in bacterial abundances between the two varieties during storage, and the screening value of the Linear discriminant analysis (LDA) score was set to 4 (Zhang et al., 2013).

#### Correlational analysis for carbohydrate factors

Redundancy analysis (RDA) plots were drawn to perform RDA between carbohydrates and specific bacterial species to obtain carbohydrate factors that significantly influenced the changes across bacterial communities. Spearman correlation coefficients were calculated using the R package, ggcor (Huang et al., 2020), based on the information for differential species abundance obtained from LEfSE analysis. Finally, a correlation analysis was performed between carbohydrates and specific bacterial communities.

## Results

### Richness and diversity analyses of bacterial communities

**Figure 1:**
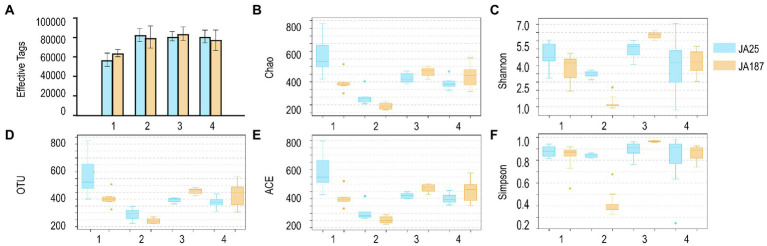
Results of the analysis of the number of effective sequences, operational taxonomic units and Alpha diversity index of bacteria on the surface of tubers of different Jerusalem artichoke resources during storage. **A**: Effective tags; **B**: Chao index; **C**: Shannon index; **D**: OTUs; **E**: ACE index; **F**: Simpson index. The numbers of replicated samples in this figure are as follows: ‘JA25’ (*n* = 24), ‘JA187’ (*n* = 24).

A total of 3,634,419 high-quality bacteria sequences were obtained from 48 samples (24 from ‘JA25’ and 24 from ‘JA187’), with each having an average of 75,717 sequences. Good’s coverage is an important metric for evaluating the sample coverage (i.e., the likelihood of sequencing the 16S rRNA PCR amplicons). As shown in Supplementary Table S1, the calculated Good’s coverage values for bacterial community abundance ranged between 0.998 and 0.999, indicating that the samples had high coverage and that the sequencing results could truly characterize the bacterial diversity on the surface of JA tubers. In addition, the α-diversity index was calculated to assess the differences in bacterial community richness and diversity among different JA varieties during the storage period. As shown in Figure 1, the changing trend for OTU species between ‘JA25’ and ‘JA187’ was the same (decreasing–increasing–decreasing). Subsequently, bacterial abundance was evaluated using Chao and ACE indices. The number of bacterial OTUs detected in the storage-tolerant variety, ‘JA25’, was higher than that in the storage-intolerant variety, ‘JA187’. Shannon and Simpson’s indices are indicators of bacterial community diversity within the samples. It is generally accepted that a high Shannon index represents greater diversity, whereas a high Simpson index represents lower diversity. Herein, the diversity of bacteria in ‘JA25’ was higher than that in ‘JA187’. Therefore, microorganisms may play a crucial role in the formation of microbial community diversity between the two varieties. Specifically, microbial community diversity was maintained in the ‘JA25’ variety. In contrast, dominant bacteria are more likely to develop in the ‘JA187’ variety, thus resulting in a reduction in diversity.

**Figure 2:**
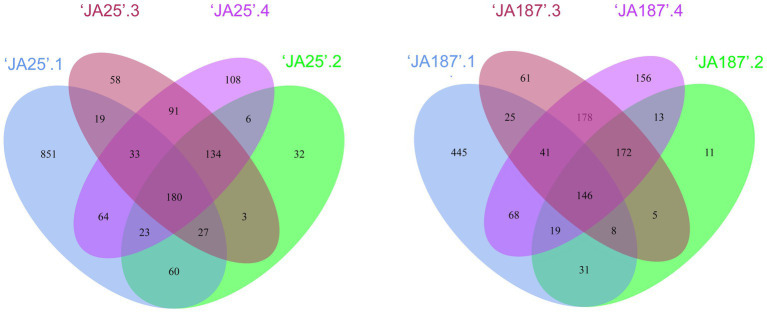
Bacterial taxa on the surface of tubers of different Jerusalem artichoke resources during storage. Venn diagram showing OTUs unique to and shared by ‘JA25’ (left) and ‘JA187’ (right) Jerusalem artichoke tuber surfaces. Each circle in the figure represents one sample. The number of overlapping areas represents the number of OTUs shared between the samples. The numbers without overlapping areas represented the number of unique OTUs in the sample.

In the Venn diagram, the circles denote different microbiomes, whereby their area of overlap represents the core. The common and unique bacteria between the two varieties were examined. A significant overlap in enriched OTUs was observed across different storage periods (Figure 2). The OTUs enriched during the fourth storage settled successfully throughout the enyire storage period. Of the 639/793 OTUs enriched in the fourth storage period, 531/637 were also enriched in the remaining three storage periods: (i.e., 531 and 637 OTUs were consistently present in the corresponding two JA varieties, during the four storage periods). Furthermore, there were differences in the number of core microbiomes, whereby ‘JA25’ had more core microbiomes in the most overlapping region than ‘JA187’, suggesting an important contribution to the enrichment of the whole microbial community.

### Main members of the bacterial community on tuber surfaces during storage

**Figure 3:**
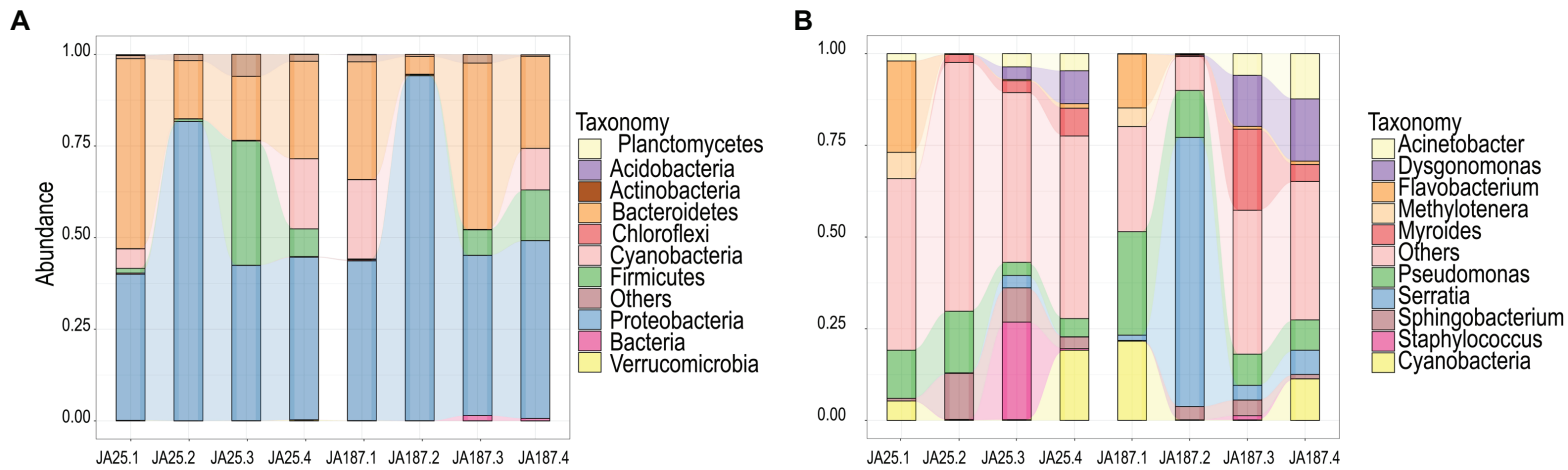
Demonstration of the composition of two Jerusalem artichoke resources at the phylum and genus level during storage. **(A)** phylum level **(B)** genus level. Each bar represents the relative abundance in each sample. Each color represents a particular phylum or genus.

The abundance of individual microbial species during storage was analyzed using the 16S rRNA sequences to elucidate the dominant bacterial composition in JA during storage. The top 10 most abundant phyla and genera are shown in Figure 3. The dominant phylum and relative abundance remained consistent between the two varieties. However, significant differences were observed in the proportion of bacteria at the genus level across the storage periods. Proteobacteria and Bacteroidetes were the dominant phyla, accounting for more than 80% of the entire community. The trend for Proteobacteria’s relative abundance between ‘JA25’ and ‘JA187’ was the same (increasing and then decreasing), reaching its peak during the second storage and then decreasing, and maintaining a constant level from the third to the fourth storage periods. The relative abundance of Bacteroidetes exhibited an overall decreasing trend during the storage of ‘JA25’, i.e., a decrease in the second period (lowest), followed by a marginal increase in the third and fourth storage periods. The pattern of variation in the relative abundance of this phylum during the storage of the ‘JA187’ variety was consistent with the observed pattern of microbial diversity from the beginning till the end of storage (Figure 3A). The abundance of unclassified fungal genera was significantly higher in the ‘JA25’ variety relative to the ‘JA187’ variety throughout the storage period. *Flavobacterium*, *Sphingobacterium*, and *Staphylococcus* were the most abundant genera at the beginning of storage in the ‘JA25’ variety, and their relative abundances decreased with the storage time. However, *Sphingobacterium* and *Staphylococcus* showed low abundances for most of the storage period in the ‘JA187’ variety. *Serratia* was the most dominant genus during storage and its relative abundance was higher than that in ‘JA25’ for the same period (Figure 3B).

### Changes in bacterial diversity during JA storage

**Figure 4:**
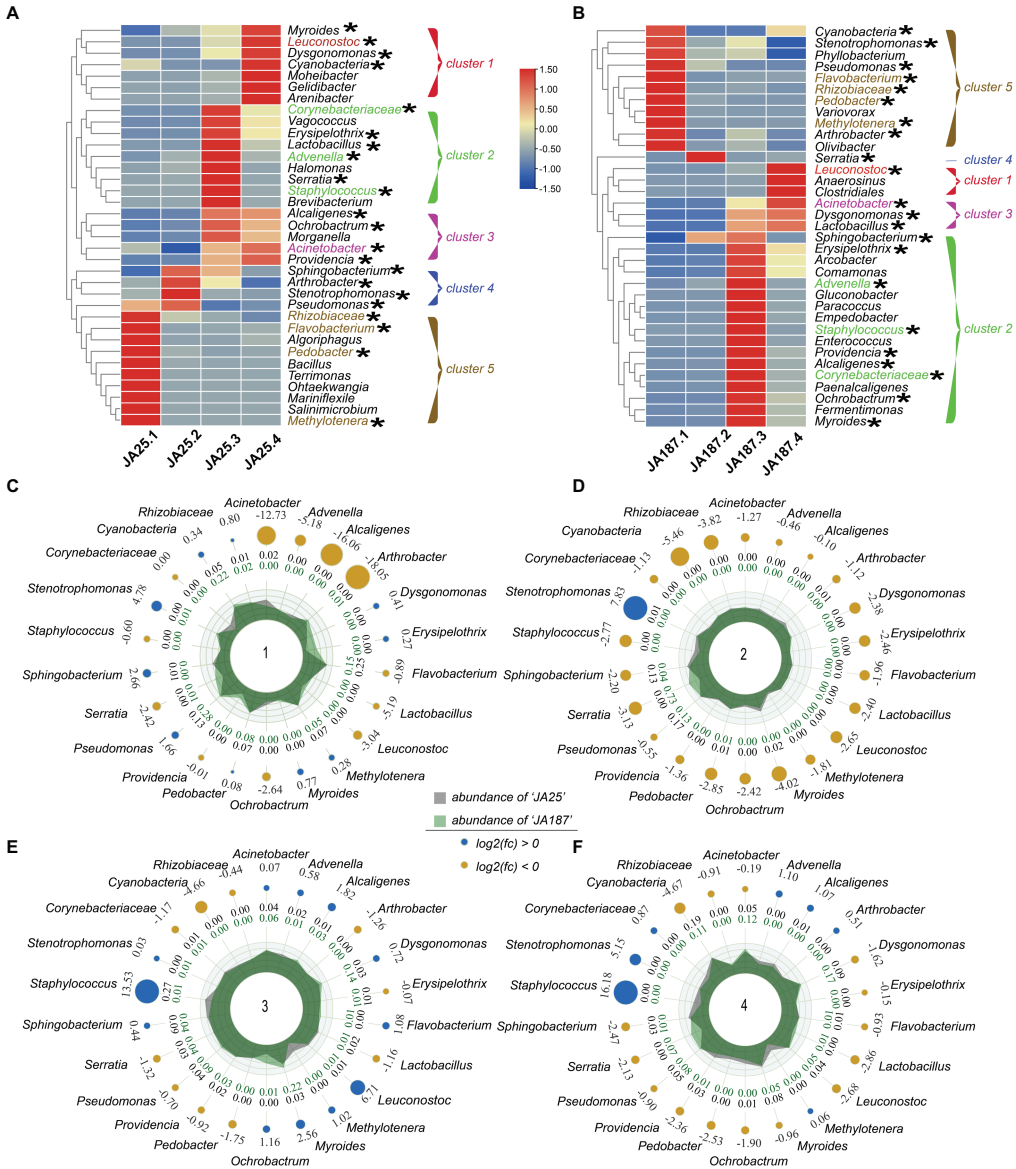
Dynamics of high-abundance bacteria in two Jerusalem artichoke resources during storage. (**A;** ‘JA25’) and (**B;** ‘JA187’) are the top 34 bacterial genus level Heatmap maps from the two resources, Cluster 1–5 indicates 5 bacterial clusters, with red and blue indicating high and low correlation, respectively. Radar plots show the abundance of a total of 22 bacteria from two chrysanthemum resources, with (**C–F**) representing four storage periods.

The top 35 genera were classified in a clustering heatmap to visualize the dynamic differences in the composition of the bacterial community (Figure 4). The changes in the abundance of bacterial communities on the tuber surfaces of the two varieties were similar across the four storage periods and could be divided into five clusters. The relative abundance of bacteria gradually increased with time in Cluster 1 but decreased in Cluster 5. Further functional analysis was performed on differential bacterial populations between the two varieties (Supplementary Table S3). Most of the genera with high relative abundances in the ‘JA187’ variety consisted of phytopathogens and carbohydrate-degrading endophytes. Phytopathogens were present in relatively low abundances in the ‘JA25’ variety and were mostly non-pathogenic. Subsequently, radar chart analysis was performed to examine the variations-in the abundances of 22 common bacteria on the surface of different JA varieties and the relative proportions of bacteria between the two varieties. The results suggested that three types of bacteria (*Acinetobacter*, Cyanobacteria, and *Staphylococcus*) were dominant among the six bacterial populations showing significant differences in their abundance. The relative abundance of *Staphylococcus,* having a biocontrol function, increased with time in the ‘JA25’ variety. In contrast, the relative abundance in the ‘JA187’ variety was low. Therefore, the differences in the function of unique bacteria and the abundances of common bacteria jointly influenced the storage process between the two JA varieties, thus proving that the differential storage characteristics between the two varieties were determined by microorganisms.

### Lefse analysis of the bacterial communities

**Figure 5:**
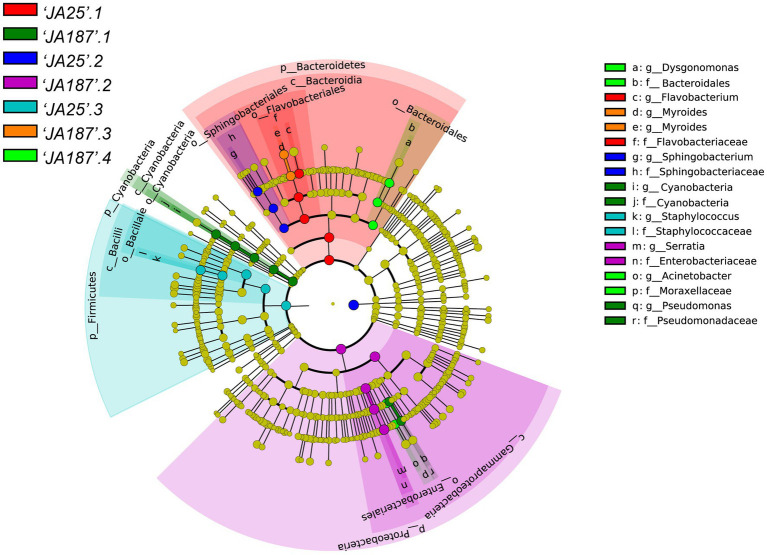
Results of LEfSe analysis of bacterial communities on the surface of tubers of different Jerusalem artichoke resources. The circle radiating from the outside to the inside represents the classification level from the phylum to the genus. Species with no significant differences are uniformly colored yellow, and the different species biomarkers follow the color of the group.

LEfSe was used to identify taxa that differed significantly in their abundance among different groups. The threshold for feature discrimination was a logarithmic LDA score of 4.0. In the LEfSe analysis, some bacterial community members exhibited a significant shift on the surface of tubers between the ‘JA25’ and ‘JA187’ varieties. At the genus level, the abundance of *Flavobacterium, Sphingobacterium*, and *Staphylococcus* increased significantly in the ‘JA25’ variety. However, six genera were present in the ‘JA187’ variety, including *Pseudomonas*, Cyanobacteria, *Serratia*, *Myroides*, *Dysgonomonas*, and *Acinetobacter*. Therefore, the storage-intolerant variety, ‘JA187’, comprised greater microorganisms during JA decay. Furthermore, the absence of ‘JA25’0.4 in the figure indicates no significant differential taxa in this group (Figure 5).

### Relationship between bacterial taxa and carbohydrates

**Figure 6:**
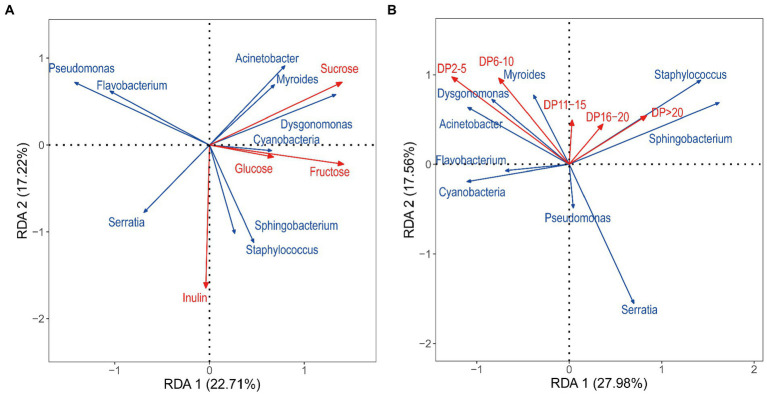
Redundancy analysis (RDA) based on bacteria OTU data with chemical parameters. (**A**): RDA between carbohydrate and bacterial communities, (**B**): RDA between the DP of inulin and bacterial communities. The correlation between carbohydrate factors and RDA axes is represented by the length and angle of arrows.

RDA was used to analyze the correlation between bacterial communities and carbohydrate factors, with the first two coordinate axes cumulatively explaining 60.93% of the variation in the microbial community structure. Based on the length of the arrow for each carbohydrate factor, inulin, sucrose, fructose, DP2–5, and DP6–10 were finally identified as key factors influencing the distribution of microbial communities in that order, whereas glucose, DP11–15, DP16–20, and DP > 20 had a little effect on species distribution (Figure 6). Specifically, Cyanobacteria, *Sphingobacterium*, *Staphylococcus*, and *Serratia* correlated positively with inulin; *Acinetobacter*, *Myroides*, and *Dysgonomona*s correlated positively with sucrose. The RDA revealed a relative aggregation of the positions of *Myroides* and *Dysgonomonas*. A positive correlation between the two with glucose and fructose was observed. Cyanobacteria correlated positively with all four sugars. *Seudomonas* and *Flavobacterium* correlated negatively with glucose and fructose. DP2–5 and DP6–10 correlated positively with the distribution of *Acinetobacter*, *Myroides*, and *Dysgonomona*s; *Myroides* correlated positively with all five DPs.

### Carbohydrate drivers of bacterial community composition

**Figure 7:**
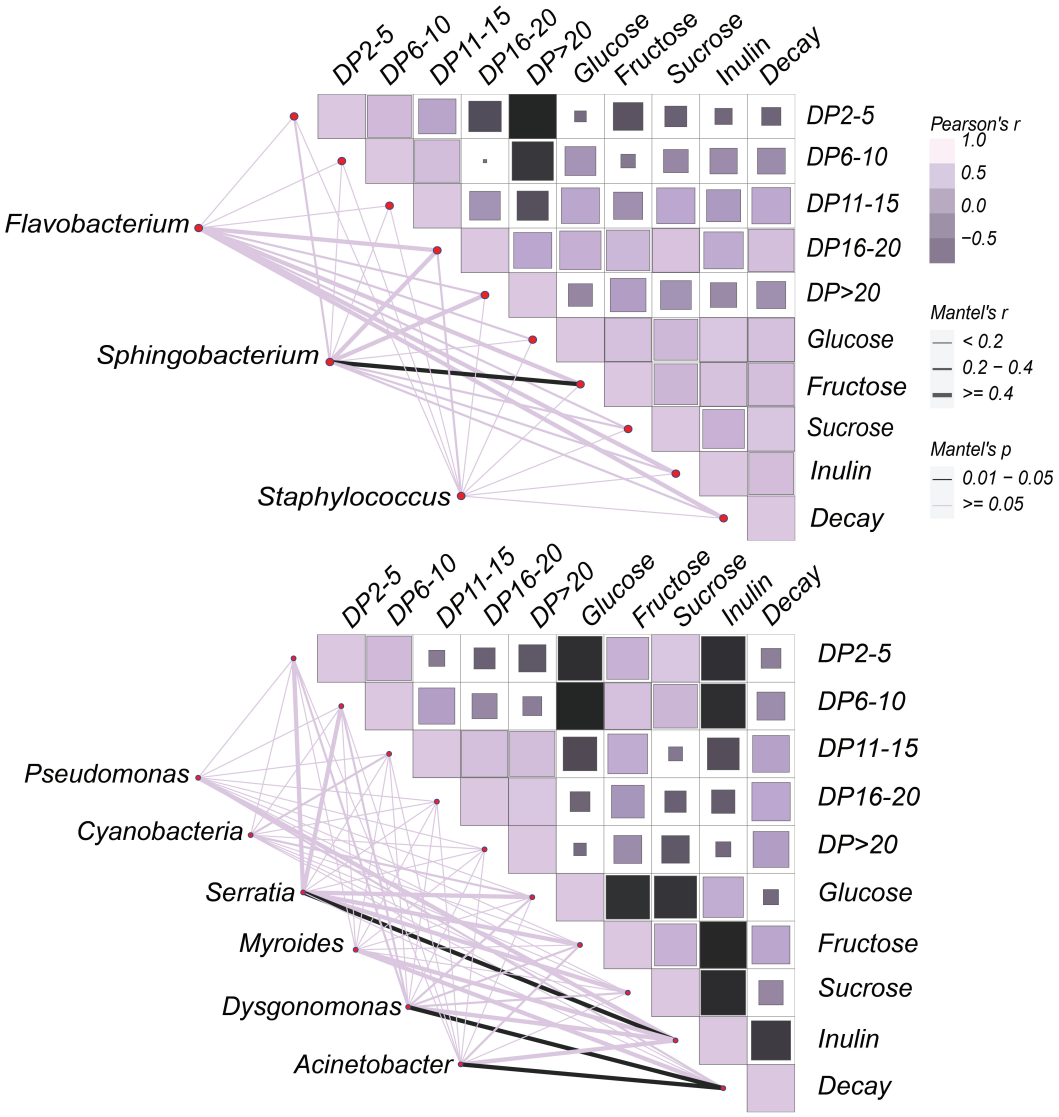
Correlation between bacterial communities and carbohydrates on the surface of Jerusalem artichoke tubers during storage. The horizontal and vertical axes of the heat map on the right side represent the various types of carbohydrate factors that respond to or interact with the community, respectively. The color of the block in each square of the heat map indicates a positive or negative correlation with carbohydrate factors, and its size indicates the absolute value of the correlation coefficient. The shading from dark purple to light purple indicates a positive correlation gradient from low to high, and the shading from dark purple to black represents a negative correlation gradient from low to high. On the left side is the bacterial data obtained from LEfSe analysis, which are linked to each carbohydrate factor data one by one through connecting lines. The edge width corresponds to the R value and edge color denotes the statistical significance.

Carbohydrates exert a strong impact on the microbial community on the surface of JA tubers. Therefore, correlations between the carbohydrate variable matrix and the species abundance matrix were visualized by Mantel test statistics (Figure 7) to identify the main carbohydrate drivers in the dataset, to facilitate the understanding of their contribution to community formation. Overall, fructose and inulin were the strongest drivers of the community formation on the tuber surface. The positive correlation between degree of polymerization (DP) of inulin-fructans and decay gradually increased with increasing DP values. Fructans with a high degree of polymerization accelerated the decay of JA tubers. In the ‘JA187’ variety, *Dysgonomonas* and *Acinetobacter* showed a strong correlation with decay, thus suggesting that bacteria associated with decay rates may be directly involved in the degradation of highly polymerized fructans. Additionally, a significant negative correlation between decay and inulin was observed, whereas *Serratia* showed a highly significant positive correlation with inulin, suggesting its antagonistic role against pathogenic bacteria on the surface of stored JA tubers. Furthermore, a strong positive correlation between decay and fructose was observed in the ‘JA25’ variety. Nevertheless, there was also a strong positive correlation between *Sphingobacterium* and fructose, indicating its inhibitory effect on the decomposition of fructose during storage. In addition, the positive correlation with inulin gradually increased with the increase in fructan within DP < 20, reaching a maximum between16-20, indicating that the fructans present in the tubers of the ‘JA25’ variety generally showed a high degree of polymerization. In the ‘JA187’ variety, the negative correlation with inulin gradually increased with decreasing DP values and reached a maximum between 2 and 5, indicating that fructans in the tubers were mainly present in a state of low polymerization. Therefore, the differences in the carbohydrate metabolite patterns may lead to the differential storage characteristics among different genotypes of JA varieties.

## Discussion

The expansion of stolons in JA forms tubers, wherein carbohydrates are stored in the form of an inulin (Li et al., 2017). Similar to rhizospheric microbes, those attached to the tuber surface are influenced to some extent by plant genetics (Walters et al., 2018). Herein, we demonstrated that selection for crop genotypes drives changes in the recruitment of the plant microbiome using two differential storage-tolerant JA varieties. At the beginning of storage, microbial communities on the tuber surface differed only in their abundance, suggesting homogeneity of the rhizospheric microbiome during the recruitment process. However, the two JA genotypes remained highly heterogeneous for carbohydrates and metabolites. Consequently, the microbial community on the tuber surface changed dramatically during storage. Several studies have confirmed that heterogeneity of crop genotypes and selection effects exert a significant impact on the assembly of the microbial community (Schmidt et al., 2020; Favela et al., 2021). Therefore, further studies are needed to determine changes in pre-harvest bacterial communities on the tuber surface to validate the effects of plant genotype on the recruitment behavior of bacteria on the surface of post-harvest tubers.

JA needs to be stored for commercial processing, especially in the Qinghai–Tibet Plateau region, where the harvesting period is concentrated in early October. Early harvesting results in insufficient tuber yield and fructan content for commercialization. However, harvesting at ground temperatures below 0°C causes the tubers to freeze, and re-thawing during intensive processing results in significant sugar loss and increased decay rates. This challenge is not confined to the alpine regions. In hot areas of Thailand, the environmental factors pose challenges in the processing of JA (Jirayucharoensak et al., 2018). Therefore, storage is the only solution to relieve the processing pressure and the most convenient and easy way to preserve germplasm resources for asexually propagated crops. Research on microbial disease infestation during JA storage has focused on fungi, including *Botrytis*, *Aspergillus*, *Fusarium*, *Rhizopus*, and *Pennicillum,* which cause the decay of JA tubers, premature germination, and decrease the fructan quality during the storage (Kosaric et al., 1984; Jin et al., 2013; Yang et al., 2020). The surface of harvested and stored crops enriches with a large population of microorganisms, including bacteria, filamentous fungi, and yeasts, either as epiphytes or endophytes (Droby et al., 2016). At present, there is a lack of knowledge on the microbial variability and diversity of tuber crops, including JA that is stored for overwintering. The residual soil attached to the tuber surfaces after harvesting is the main contributor to the bacterial community diversity during storage, as evidenced by the phylum-level observations between the two stored varieties’ tubers during the initial period of storage. Apart from some differences in abundance, the degree of differences at the species level was not high. Indicators of microbial diversity of ‘JA187’ were significantly lower in the first two storage periods as compared to the storage-tolerant variety. Disease outbreaks in plants are often correlated with shifts in the microbiome composition, resulting in microbial dysbiosis (Berg et al., 2017). The abundance of *Serratia* in the perishable variety, ‘JA187’, changed dramatically especially during the second storage period, leading to a decrease in the abundance of bacterial communities. *Serratia rubidaea* and *Serratia plymuthica* isolated from onion corms are the main causal agent of tulip bulbs (Kowalska et al., 2011; Stoyanova et al., 2012). In contrast, *Serratia plymuthica* plays an antagonistic role against fungal diseases during the storage of potato tubers (Gould et al., 2008; Czajkowski et al., 2012; Maciag et al., 2020). Most of the potential biocontrol agents are screened by *in vitro* antagonism activity against the pathogen. However, there is a long and intense debate on the screening assay (Berg et al., 2017). The artificial environment leads to no or only a very low *in vitro* antagonism (Adesina et al., 2009). However, bacteria that exhibit antagonistic effects in the *in vitro* experiments exert opposite effects due to the lack of community support (Rybakova et al., 2016). Pathogen infestation and spoilage may often not be caused by a single organism but is likely to result from the interplay of individual members of the microbial community in crops. Microbes enriched in unrotten samples primarily belong to Cyanobacteria, *Staphylococcus*, and *Staphylococcaceae*, which could act as potential biocontrol agents against rot (Prasanna et al., 2015; Cebrián et al., 2020; Lorenzini and Zapparoli, 2020; Shah et al., 2021). We speculated that this effect could originate directly from the impact of the biocontrol agents on the composition of the microbiota or indirectly from their impact on a pathogen. Treatment of ginger (*Zingiber officinale* Roscoe) with biological control agents using *Bacillus* and *Trichoderma*, significantly alter the structure and diversity of bacterial communities on its surface (Huang et al., 2021).

In the plant taxa, there is a close association between seeds and bacteria. Tuber crops develop and mature in the soil, and microorganisms on the surface of the tuber during this process have a distinct relationship with the environment. In particular, microorganisms attached to the surface of root crops have been observed, including *Staphylococcus*, *Pseudomonas*, and *Actinobacteria* (Buchholz et al., 2019, 2021; Shi et al., 2019). This finding is consistent with the microbial species on the surface of the JA tubers during storage herein, which may be attributed to the wide distribution of these bacteria that genera-level in soils worldwide (Fierer et al., 2012), and thus, is the genus of the most likely taxa that encounter tubers during the developmental stages and beyond (Nelson, 2018). In addition, a significant difference in tuber bacterial communities was observed between the two genotypes during dormancy (the second and third storage periods), indicating the continuous dynamics of bacterial communities during dormancy. Interestingly, changes in microbial communities observed during the storage of potatoes and sugar beets are independent of crop genotypes (Kusstatscher et al., 2019; Buchholz et al., 2021). This result is not quite consistent with our findings, which may be attributed to the differences in carbohydrates or a selective relationship between bacterial communities and carbohydrates due to the chemical specificity of fructans. The Baas Becking hypothesis propounds that ‘everything is everywhere but the environment selects.’ Therefore, environmental selection is the primary evolutionary driver in a gradient-differentiated environment, regardless of the size of the community. Indeed, natural selection has dominated changes in communities. Many microbial community studies have correspondingly only attempted to evaluate the role of selection in community assemblage. Overall, there has been less focus on evaluating whether microbial communities may differentiate as a consequence of various neutral processes (Hanson et al., 2012; Morrison-Whittle and Goddard, 2015).

The human gut microbiota encodes a huge diversity of enzymes for the digestion of all components of plant polysaccharides including inulin. A study on inulin and arabinoxylan-oligosaccharides as carbon sources for simulation experiments shows that the complexity of the carbon source structure maintains a greater microbial diversity (Chung et al., 2019). The relationships between carbohydrate structural complexity and sustained diversity may be a fundamental property underlying the carbohydrate-microbiome interactions (Yao et al., 2020). The results of the correlational analysis revealed significant associations between continuous changes in carbohydrate and bacterial communities, with positive or negative correlations between decay and several carbohydrates. These data suggested that changes in carbohydrate structure could alter the microbial communities that consume specific carbohydrates. In a continuously changing environment, decay consistently shows a positive correlation with degree of polymerization (DP) of inulin-fructans, and high-DP inulin accelerates the decay of JA tubers. Therefore, decay-related bacteria may be directly involved in the decomposition of degree of polymerization (DP) of inulin-fructans, as evidenced b a significant correlation of *Dysgonomonas* and *Acinetobacter* in storage-intolerant JA varieties, ‘JA187’, with decay. *Dysgonomonas* is found in the gut of many insects (McManus et al., 2018; Jang and Kikuchi, 2020) and are commonly cultured on complex media containing blood, peptone, tryptone, yeast, or plants under anaerobic conditions (Bridges and Gage, 2021). We hypothesized that bacteria belonging to the genus, *Dysgonomonas,* play a driving role in the decay of JA tubers. This is one of the few studies on plants wherein the function of *Dysgonomonas* has been elucidated, and there may be an unknown important role in the degradation and utilization of inulin. *Acinetobacter* is capable of producing high amounts of inulinase (Muslim et al., 2015), and inulin is an excellent substrate. Therefore, *Acinetobacter* can spearhead the catabolic conversion of inulin during storage of JA tubers, as monosaccharides produced by the degradation of inulin are the best culture substrates for several microorganisms. *Serratia* was mainly enriched in ‘JA187’ but it cannot directly ferment inulin (Gavini et al., 1979). In addition, 10% inulin content has a good inhibitory effect on multiple bacteria including *Serratia* (Salman, 2009). Results of the correlational analysis revealed a highly significant positive correlation of *Serratia* with inulin during storage; an opposite trend was observed with decay rate, confirming that *Serratia* played an antagonistic role against pathogenic bacteria on the surface of stored JA tubers. However, we did not observe the aforementioned association between bacteria and carbohydrates in storage-tolerant varieties, and decay exhibited a positive correlation with inulin. These findings suggested that crop genotypes cause differential microbial community-related selection effects, as environmental conditions for tuber storage and pre-harvest planting soil conditions were consistent. *Sphingobacterium* showed a positive correlation with fructose in storage-tolerant varieties, which may be attributed to the inhibition of fructose catabolite utilization. *Sphingobacterium* aerobic bacterium is capable of suppressing *Fusarium graminearum*, *Exserohilum turcicum*, *Pythium aphanidermatum*, and *Cochliobolus sativus.* The strain of *Sphingobacterium* harbors sets of genes responsible for the production of 2,3-butanediol and salicylic acid, which can elicit induced systemic resistance in the host plant (Xu et al., 2020; Sahu et al., 2021). Information on the dynamics and diversity of microbiota in stored JA may be useful for developing a new paradigm in postharvest biocontrol based on the construction of synthetic microbial communities that provide superior pathogen control strategiess.

## Conclusion

In Jerusalem artichoke tuber, the inulin-type fructans serve as carbohydrate reserve, inulin are one source of soluble dietary fibers. Currently, very few studies have been reported on the association of inulin with bacterial communities during JA storage, especially in fructane-based tuber crops. The analysis of the results in this study proved that *Flavobacterium*, *Sphingobacterium*, *Staphylococcus*, *Dysgonomonas*, *Acinetobacter* and *Serratia* assumed an important role in the storage process. Both crop genotype and carbohydrate structure affected the bacterial community composition.

## Data availability statement

The data presented in the study are deposited in the https://www.ncbi.nlm.nih.gov/ repository, accession number PRJNA822494.

## Author contributions

GD: conceptualization, methodology, formal analysis, investigation, data curation, writing–original draft, and visualization. ZS: visualization and writing–review and editing. SB: investigation, methodology, and data curation. QZ: resources, data curation, and funding acquisition. SY: conceptualization, methodology, resources, data curation, writing–review and editing, supervision, funding acquisition, and project administration. All authors contributed to the article and approved the submitted version.

## Funding

This study was financially supported by Key Laboratory Project of Qinghai Science & Technology Department (Grant reference: 2020-ZJ-Y02), Qinghai key research and development conversion project (Grant reference: 2022-NK-117), and National Natural Science Foundation of China (31760600).

## Conflict of interest

The authors declare that the research was conducted in the absence of any commercial or financial relationships that could be construed as a potential conflict of interest.

## Publisher’s note

All claims expressed in this article are solely those of the authors and do not necessarily represent those of their affiliated organizations, or those of the publisher, the editors and the reviewers. Any product that may be evaluated in this article, or claim that may be made by its manufacturer, is not guaranteed or endorsed by the publisher.

## Footnotes

^1^ http://ccb.jhu.edu/software/FLASH/

^2^ http://www.drive5.com/uparse/

^3^ http://qiime.org/scripts/split_libraries_fastq.html
