# Supplementary material for: Diversity of bacterial community in Jerusalem artichoke (Helianthus tuberosus L.) during storage is associated with the genotype and carbohydrates
Source: Front Microbiol. 2022 Sep 15;13:986659. doi: 10.3389/fmicb.2022.986659 (PMC9520535; doi:10.3389/fmicb.2022.986659)
Supplement: Supplementary file 1 [file Table_1.DOCX]

Table S1 HPLC determination of carbohydrates in Jerusalem artichoke during storage

| Samples | Glucose  (mg/g DW) | Fructose  (mg/g DW) | Sucrose  (mg/g DW) | Inulin  (mg/g DW) |
| --- | --- | --- | --- | --- |
| ‘JA25’.1.1 | 0.372887328 | 1.168735009 | 1.654316559 | 394.2635366 |
| ‘JA25’.1.2 | 0.389552921 | 1.383097477 | 1.195075744 | 439.7453785 |
| ‘JA25’.1.3 | 0.309030471 | 1.234570181 | 1.71690754 | 479.4539898 |
| ‘JA25’.1.4 | 0.35419571 | 1.354338962 | 1.383451158 | 387.5908934 |
| ‘JA25’.1.5 | 0.068345681 | 1.146375804 | 2.10346857 | 450.208848 |
| ‘JA25’.1.6 | 0.291833408 | 1.074117337 | 1.525491903 | 384.1723853 |
| ‘JA25’.2.1 | 0.163166269 | 1.251647798 | 1.374525509 | 436.2793034 |
| ‘JA25’.2.2 | 0.231065516 | 1.753456299 | 2.247776423 | 396.9315964 |
| ‘JA25’.2.3 | 0.23211557 | 1.692792382 | 2.167967961 | 351.4480519 |
| ‘JA25’.2.4 | 0.205757908 | 1.5950662 | 1.943527773 | 409.5310658 |
| ‘JA25’.2.5 | 0.289317787 | 1.564086635 | 1.990447501 | 367.0154943 |
| ‘JA25’.2.6 | 0.661823101 | 4.393027439 | 2.356423472 | 336.0365283 |
| ‘JA25’.3.1 | 0.628057077 | 4.419584093 | 2.392484764 | 329.2353129 |
| ‘JA25’.3.2 | 0.606534175 | 4.199503502 | 2.385279471 | 326.0939939 |
| ‘JA25’.3.3 | 0.109861496 | 1.240336276 | 1.350182831 | 415.486613 |
| ‘JA25’.3.4 | 0.610241577 | 5.028050945 | 2.229872889 | 213.4092986 |
| ‘JA25’.3.5 | 0.597302712 | 4.495282069 | 2.269326136 | 294.0759802 |
| ‘JA25’.3.6 | 0.6264981 | 4.662340497 | 2.127403796 | 314.5687024 |
| ‘JA25’.4.1 | 0.735059589 | 4.121243884 | 2.431140519 | 337.5422751 |
| ‘JA25’.4.2 | 0.64594666 | 4.118802168 | 2.784314818 | 334.1433457 |
| ‘JA25’.4.3 | 0.597789087 | 4.12059148 | 2.4633432 | 215.3760144 |
| ‘JA25’.4.4 | 0.466480706 | 3.002947808 | 2.589500261 | 357.9691647 |
| ‘JA25’.4.5 | 0.690715712 | 4.498673606 | 2.805948111 | 268.3670026 |
| ‘JA25’.4.6 | 0.415888037 | 2.890072436 | 2.218707992 | 393.8396309 |
| ‘JA187’.1.1 | 0.992004123 | 5.742777991 | 3.630012189 | 138.4873223 |
| ‘JA187’.1.2 | 0.826626941 | 4.730497458 | 3.812993209 | 173.7595744 |
| ‘JA187’.1.3 | 1.121676222 | 5.741669865 | 3.298279645 | 179.5605893 |
| ‘JA187’.1.4 | 0.91370096 | 5.193392018 | 3.847142608 | 193.4478999 |
| ‘JA187’.1.5 | 0.89130516 | 4.812613931 | 3.358241337 | 285.9240292 |
| ‘JA187’.1.6 | 0.65247246 | 4.001551856 | 3.310074874 | 251.6083624 |
| ‘JA187’.2.1 | 1.251982864 | 4.527086731 | 2.263405885 | 222.9070172 |
| ‘JA187’.2.2 | 0.938422341 | 4.037141418 | 1.907664983 | 283.5157678 |
| ‘JA187’.2.3 | 1.454472718 | 5.169574499 | 2.459519415 | 181.774467 |
| ‘JA187’.2.4 | 1.015182632 | 3.146812338 | 1.831206686 | 266.8065054 |
| ‘JA187’.2.5 | 1.063581782 | 4.859879593 | 2.019432352 | 244.5801077 |
| ‘JA187’.2.6 | 1.220371707 | 5.158133455 | 2.326435661 | 385.6838714 |
| ‘JA187’.3.1 | 1.080166849 | 5.597076178 | 3.347943583 | 262.0128292 |
| ‘JA187’.3.2 | 0.983387876 | 5.504636381 | 3.50045969 | 197.8781784 |
| ‘JA187’.3.3 | 1.150079237 | 4.65151348 | 4.671854431 | 215.7247279 |
| ‘JA187’.3.4 | 1.051357985 | 5.499537081 | 3.884499391 | 206.4405252 |
| ‘JA187’.3.5 | 0.647128777 | 3.368073012 | 4.101730803 | 167.7169209 |
| ‘JA187’.3.6  ‘JA187’.4.1 | 1.101825034  0.977992656 | 6.375134318  6.003902427 | 4.219324395  3.194086714 | 141.2758291  267.2037592 |
| ‘JA187’.4.2 | 0.719244347 | 4.55527919 | 3.497865227 | 287.8769221 |
| ‘JA187’.4.3 | 0.702569091 | 4.42453948 | 3.589939056 | 283.0716487 |
| ‘JA187’.4.4 | 1.257529472 | 6.907529022 | 3.504767543 | 271.5706703 |
| ‘JA187’.4.5 | 0.959259164 | 6.07715389 | 3.5419502 | 199.0945331 |
| ‘JA187’.4.6 | 0.848906783 | 5.4175981 | 3.426418248 | 204.1752115 |

Continued Table

| Samples | HPLC  graph |
| --- | --- |
| ‘JA25’.1.1 | 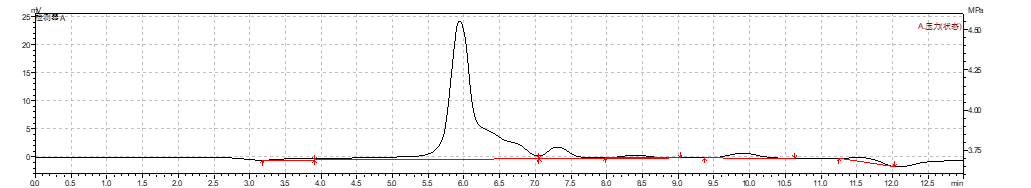 |
| ‘JA25’.1.2 |  |
| ‘JA25’.1.3 |  |
| ‘JA25’.1.4 |  |
| ‘JA25’.1.5 |  |
| ‘JA25’.1.6 |  |
| ‘JA25’.2.1 |  |
| ‘JA25’.2.2 |  |
| ‘JA25’.2.3 |  |
| ‘JA25’.2.4 |  |
| ‘JA25’.2.5 |  |
| ‘JA25’.2.6 |  |
| ‘JA25’.3.1 |  |
| ‘JA25’.3.2 |  |
| ‘JA25’.3.3 |  |
| ‘JA25’.3.4 |  |
| ‘JA25’.3.5 |  |
| ‘JA25’.3.6 |  |
| ‘JA25’.4.1 |  |
| ‘JA25’.4.2 |  |
| ‘JA25’.4.3 |  |
| ‘JA25’.4.4 |  |
| ‘JA25’.4.5 |  |
| ‘JA25’.4.6 |  |
| ‘JA187’.1.1 |  |
| ‘JA187’.1.2 |  |
| ‘JA187’.1.3 |  |
| ‘JA187’.1.4 |  |
| ‘JA187’.1.5 |  |
| ‘JA187’.1.6 |  |
| ‘JA187’.2.1 |  |
| ‘JA187’.2.2 |  |
| ‘JA187’.2.3 |  |
| ‘JA187’.2.4 |  |
| ‘JA187’.2.5 |  |
| ‘JA187’.2.6 |  |
| ‘JA187’.3.1 |  |
| ‘JA187’.3.2 |  |
| ‘JA187’.3.3 |  |
| ‘JA187’.3.4 |  |
| ‘JA187’.3.5 |  |
| ‘JA187’.3.6 |  |
| ‘JA187’.4.1 |  |
| ‘JA187’.4.2 |  |
| ‘JA187’.4.3 |  |
| ‘JA187’.4.4 |  |
| ‘JA187’.4.5 |  |
| ‘JA187’.4.6 |  |

Note: Linear regression equation of sucrose fructose glucose standard. Sucrose: y = 28715x + 1450.2(R2 = 0.997); Glucose: y = 31046x + 112.24(R2 = 1); Fructose: y = 20846x - 379.85(R2 = 1).

Table S2 Biodiversity analysis of 16S rRNA sequences in bacterial V4 region in Jerusalem artichoke during storage.

| Samples | OTUs | Chao | ACE | Simpson | Shannon | Goods coverage |
| --- | --- | --- | --- | --- | --- | --- |
| ‘JA25’.1 | 571 | 569.303 | 585.843 | 0.876 | 4.579 | 0.998 |
| ‘JA187’.1 | 405 | 397.514 | 404.978 | 0.797 | 3.810 | 0.999 |
| ‘JA25’.2 | 288 | 302.677 | 307.800 | 0.838 | 3.481 | 0.998 |
| ‘JA187’.2 | 241 | 240.210 | 252.471 | 0.442 | 1.827 | 0.999 |
| ‘JA25’.3 | 392 | 423.707 | 420.210 | 0.888 | 4.933 | 0.998 |
| ‘JA187’.3 | 459 | 464.037 | 470.789 | 0.964 | 5.824 | 0.999 |
| ‘JA25’.4 | 376 | 395.863 | 399.076 | 0.746 | 3.973 | 0.998 |
| ‘JA187’.4 | 431 | 437.233 | 453.153 | 0.857 | 4.184 | 0.999 |

Note: Operational taxonomic units (OTUs) were the classified operational units; the diversity indexes included (Chao) representing the species richness, abundance-based coverage (ACE) indicating the uniformity; (Simpson) and (Shannon) defining as the diversity index. Good’s coverage was calculated using QIIME software at a level of similarity of 97%. Noted 1-4: The four periods of storage; ‘JA25’: Resource JA25; ‘JA187’: Resource JA187.

Table S3 Functional summary of the differential bacterial groups on the surface of tubers

| Resources | Phylum | Family | Genus | Function | References |
| --- | --- | --- | --- | --- | --- |
| ‘JA25’ | Bacteroidetes | unidentified_Flavobacteriales | Moheibacter | Model strains, pathogenic objects without | http://www.nimr.org.cn/Column.asp?Column_ID=334997597&Column=334997597 |
|  | Bacteroidetes | Flavobacteriaceae | Gelidibacter | Potentially denitrifying bacteria, pathogenic objects without | http://www.nimr.org.cn/Column.asp?Column_ID=334997597&Column=334997597 |
|  | Bacteroidetes | Flavobacteriaceae | Arenibacter | Five new phenethylamine (PEA) derivatives (1–5) were isolated from the strain of Arenibacter nanhaiticus sp. Of these compounds, 5 showed weak antimicrobial activity against Staphylococcus aureus and Bacillus subtilis | https://wwwnature.53yu.com/articles/ja201365 |
|  | Firmicutes | Enterococcaceae | Vagococcus | Vagococcus fluvialis showed good protection against Vibrio anguillarum 975-1 in Dicentrarchus labrax vivo,this strain to be a good candidate for use as a future probiotic in aquaculture. | https://www.sciencedirect.com/science/article/pii/S0378113511005074 |
|  | Actinobacteria | Brevibacteriaceae | Brevibacterium | Presenting potential for use as a biopreservative in food systems. | https://sfamjournals.onlinelibrary.wiley.com/doi/full/10.1046/j.1365-2672.2002.01490.x |
|  | Proteobacteria | Enterobacteriaceae | Morganella | Conditionally pathogenic bacteria, not fermenting sucrose, fermenting glucose | http://www.nimr.org.cn/Column.asp?Column_ID=334997597&Column=334997597 |
|  | Bacteroidetes | Cyclobacteriaceae | Algoriphagus | Algoriphagus sp. has the ability to degrade polysaccharides and other macromolecules | https://journals.asm.org/doi/full/10.1128/JB.01421-10 |
|  | Firmicutes | Bacillaceae | Bacillus | The large majority of Bacillus species are harmless saprophytes. | https://cidta.usal.es/cursos/enfermedades/modulos/libros/UNIDAD5/Bacillus%20baron.pdf |
|  | Bacteroidetes | Chitinophagaceae | Terrimonas | Glucose, lactose, etc. can be used | http://www.nimr.org.cn/Column.asp?Column_ID=334997597&Column=334997597 |
|  | Bacteroidetes | Microscillaceae | Ohtaekwangia | Antimicrobial Activity | https://journal.ipb.ac.id/index.php/hayati/article/view/39212 |
|  | Bacteroidetes | Flavobacteriaceae | Mariniflexile | Potential plant-growth-promoting micro-organisms | https://www.mdpi.com/2077-0472/8/12/184 |
|  | Bacteroidetes | Flavobacteriaceae | Salinimicrobium | Denitrifying bacteria, soil salt-tolerant bacteria, salinophilic bacteria | https://www.sciencedirect.com/science/article/abs/pii/S0341816221000710 |
|  | Proteobacteria | Halomonadaceae | Halomonas | Halotolerant (also described as slight to moderate halophiles) | https://www.sciencedirect.com/science/article/abs/pii/S0923250806001288 |
| ‘JA187’ | Proteobacteria | Burkholderiaceae | Variovorax | Antimicrobial activities | https://scholarworks.lib.csusb.edu/etd/584/ |
|  | Bacteroidetes | Sphingobacteriaceae | Olivibacter | A hydrocarbon-degrading bacterium, there is no evidence in the literature for the potential role of Olivibacter in suppressing pathogens. | https://www.microbiologyresearch.org/content/journal/ijsem/10.1099/ijs.0.026641-0  https://wwwnature.53yu.com/articles/s41522-021-00204-9 |
|  | Firmicutes | Veillonellaceae | Anaerosinus | Significant ability to ferment carbohydrates | https://pesquisa.bvsalud.org/portal/resource/pt/vtt-204587 |
|  | Bacteroidetes | unidentified_Bacteroidales | Fermentimonas | Fermentimonas is an anaerobic acetogen and utilizes carbohydrates producing H2, CO2, and acid | https://www.sciencedirect.com/science/article/abs/pii/S0960852421019738 |
|  | Proteobacteria | Rhizobiaceae | Phyllobacterium | Using a variety of sugars or salts of organic acids as carbon sources. | https://onlinelibrary.wiley.com/doi/abs/10.1002/9781118960608.gbm00840 |
|  | unidentified_Bacteria | unidentified_Campylobacterales | Arcobacter | Human, animal pathogenic bacteria | https://sfamjournals.onlinelibrary.wiley.com/doi/full/10.1111/j.1472-765X.2005.01841.x |
|  | Proteobacteria | Burkholderiaceae | Comamonas | Hydrolysable gelatin, starch, Tween 80, H2S production | http://www.nimr.org.cn/Column.asp?Column_ID=334997597&Column=334997597 |
|  | Proteobacteria | Acetobacteraceae | Gluconobacter | Gluconobacter strains flourish in sugary niches and are capable of causing bacterial rot of apples and pears accompanied by various shades of browning. | https://www.caister.com/backlist/jmmb/v/v3/v3n3/15.pdf |
|  | Proteobacteria | Rhodobacteraceae | Paracoccus | Anti-phytopathogenic fungi using D-fructose, glucose, etc. | http://www.nimr.org.cn/Column.asp?Column_ID=334997597&Column=334997597 |
|  | Bacteroidetes | unidentified_Flavobacteriales | Empedobacter | Several carbohydrates are oxidized | https://onlinelibrary.wiley.com/doi/abs/10.1002/9781118960608.gbm00309 |
|  | Firmicutes | Enterococcaceae | Enterococcus | Enterococcus could utilize glucose, lactose, and sucrose. | https://www.sciencedirect.com/science/article/abs/pii/S037811191930856X |
|  | Proteobacteria | Burkholderiaceae | Paenalcaligenes | Fermented mattress material for pig farming | http://www.nimr.org.cn/Column.asp?Column_ID=334997597&Column=334997597 |
|  | Firmicutes | unidentified_Clostridiales | Clostridiales | butyrate producers, A decrease of butyrate has been proved to be positively correlated with diabetes. | https://www.hindawi.com/journals/mi/2021/5110276/ |
